# Functional recovery of a 41-year-old quadriplegic spinal cord injury patient following multiple intravenous infusions of autologous adipose-derived mesenchymal stem cells: a case report

**DOI:** 10.3389/frtra.2023.1287508

**Published:** 2023-12-07

**Authors:** Ridhima Vij, Hosu Kim, Hyeonggeun Park, Thanh Cheng, Djamchid Lotfi, Donna Chang

**Affiliations:** ^1^ Clinical Research, Hope Biosciences Research Foundation, Sugar Land, TX, United States; ^2^ Cell Production, Hope Biosciences, Sugar Land, TX, United States

**Keywords:** adipose-derived, mesenchymal stem cells, transplantation, spinal cord injury, autologous, case report

## Abstract

Spinal cord injury (SCI) is a debilitating disease with clinical manifestations ranging from incomplete neurological deficits affecting sensory and motor functions to complete paralysis. Recent advancements in stem cell research have elucidated the therapeutic potential of mesenchymal stem cells (MSCs) for the treatment of patients with SCI. Here, we present a case of a 41-year-old quadriplegic male individual who experienced a traumatic C-5 incomplete SCI, after slipping off a boat in Florida Keys on August 4, 2017. He was diagnosed with C5–C6 Grade 2 anterolisthesis with flexion teardrop fracture of the anterior C6 with jumped facet on the right and perched facet on the left at C5–C6 with spinal canal stenosis. On September 12, 2019, an Individual Expanded Access Protocol was approved for administration of multiple infusions of autologous, adipose-derived MSCs (adMSCs) for the treatment of this quadriplegic incomplete C5-6 SCI patient. Thirty-four (34) recurrent infusions each with 200 million cells were administered, over a period of ∼2.5 years, which resulted in significant improvements in his quality-of-life as demonstrated by substantial improvements in SCIM-III (Spinal Cord Independence Measure III) scores. Additionally, electromyography/nerve conduction velocity (EMG/NCV) studies showed improvements in the patient's motor and sensory function. No safety concerns were presented, and no serious adverse events were reported during the entire course of treatment. Multiple intravenous infusions of autologous HB-adMSCs for treatment of SCI demonstrated significant enhancements in the patient's neurological function with improved quality-of-life. Further research is needed to evaluate the results of this study.

## 1. Introduction

Spinal cord injury (SCI) is a chronic neurological disorder caused by damage to the spinal cord leading to disruption of neural circuitry that may cause temporary or permanent, motor, sensory or autonomic dysfunctions. SCI is a debilitating disease affecting more than 1 million people in the United States alone, with approximately 17,000 new cases occurring every year (1). Individuals who sustain SCI suffer from varying degrees of impairments that adversely affect their quality-of-life.

The pathophysiology of SCI involves an intense inflammatory response, with elevated levels of cytokines and pro-inflammatory mediators. The instantaneous primary injury event involves mechanical trauma to the spinal cord and its components that sets into motion a local and systemic response with both acute and chronic effects. This massive biochemical cascade contributes to a wide range of pathological events that serve as a substrate to secondary degeneration involving neurotoxicity, vascular dysfunction, glial scarring, neuroinflammation, apoptosis and demyelination (2–6).

Despite substantial advancements in our understanding of the complex pathophysiology of SCI, completely restorative treatments and neuroprotective resources for SCI management are limited. However, several pre-clinical studies have demonstrated the regenerative potential of Mesenchymal Stem Cells (MSCs) in the establishment of spinal cord repair protocols (7–9). In addition to their ability to differentiate into mesodermal lines, MSCs also possess the potential to differentiate into the cells of neural lineage, including neurons and glia (10). Through their paracrine activity, MSCs have also been demonstrated to exert their regenerative effects by secreting a broad range of bioactive molecules including cytokines and growth factors that contribute to immunosuppression, apoptosis inhibition, enhanced angiogenesis, and myelination (11). Moreover, MSCs release anti-inflammatory molecules that may protect damaged tissues (12). The efficacy of MSCs in ameliorating SCI has also been shown by a few clinical trials. These studies demonstrated transplantation of MSCs resulted in motor and sensory improvements as well as improvements in the quality-of-life of patients with SCI (13, 14). In addition, MSCs derived from multiple sources have been shown to promote axonal regeneration, thereby improving both neurological function and the American Spinal Cord Injury Association (ASIA) grade of SCI patients (13, 15–17). In this case study, multiple infusions of fresh, autologous, culture-expanded, adipose-derived MSCs were administered to a 41-year-old quadriplegic male individual with an incomplete C5–C6 SCI, with an objective to improve his overall quality-of-life.

## 2. Case presentation

### 2.1. Case history

Here, we report a case of a 41-year-old male individual who experienced a traumatic C-5 incomplete SCI more than 5 years prior, on August 4, 2017, when he slipped off a boat while in the Florida Keys for a family wedding and was immediately unable to move his legs. He was diagnosed with a C5–C6 Grade 2 anterolisthesis of C5 on C6:7 mm, with flexion teardrop fracture of the anterior aspect of C6, as well as jumped facet on the right and perched facet on the left at C5–C6 with spinal canal stenosis with cord compression, as well as with aspiration and near drowning. Post-injury, the subject was immediately started on a hypothermia protocol to reduce spinal cord edema. His hospital course got complicated by aspiration pneumonia. He was discharged to Texas via air ambulance for physical rehabilitation on August 23, 2017, with a diagnosis of ASIA B C-5 SCI. He underwent anterior cervical discectomy and fusion (ACDF) of C5–C6 and reduction of jumped facets for inpatient PT/OT and finally outpatient PT/OT in Texas. At the final discharge in January 2019, muscle testing scores were compared to April 2018, which revealed no change. Also, his neurological assessment conducted at the discharge revealed normal sensation to light touch and temperature. Extraocular muscles exhibited no signs of dysfunction, and all cranial nerves (2nd through the 12th) were found to be intact. Muscle strength in the upper extremities was graded as 5/5, while in all other areas, it was noted as 0/5. His reflexes were brisk and graded as 2+ in the upper extremities and 2+ in the ankles. Additionally, the patient demonstrated an absence of rectal tone.

Functionally, this subject was able to self-propel his manual wheelchair and, self-cath his bladder with set-up, but relied on family for suppository insertion necessary for bowel movements, and was concerned about incontinence when he was out, because he could not sense the need to defecate until it was immediate. He was unable to operate a modified vehicle and was dependent on family for transportation. Since September 2017, he had required the following medications to manage spasms, pain, and bowel elimination: Baclofen (20 mg) 2 times daily, Methocarbamol (750 mg) 3 times daily, Diclofenac sodium (50 mg) 3 times daily, Senna (8.6 mg) daily prn as needed for bowel movement and Dulcolax suppository as needed if no bowel movement.

There were not many restorative treatment options available for the patient. Under an Individual Patient Expanded Access Protocol, a request was submitted to the FDA, with a primary goal to improve the quality-of-life of this quadriplegic incomplete C5-6 SCI patient. On September 12, 2019, Institutional Review Board (IRB) approved intravenous administration of multiple doses of autologous, Hope Biosciences adipose-derived MSCs (HB-adMSCs) for the treatment.

### 2.2. Isolation and culture of adipose-derived MSCs

**Figure 1:**
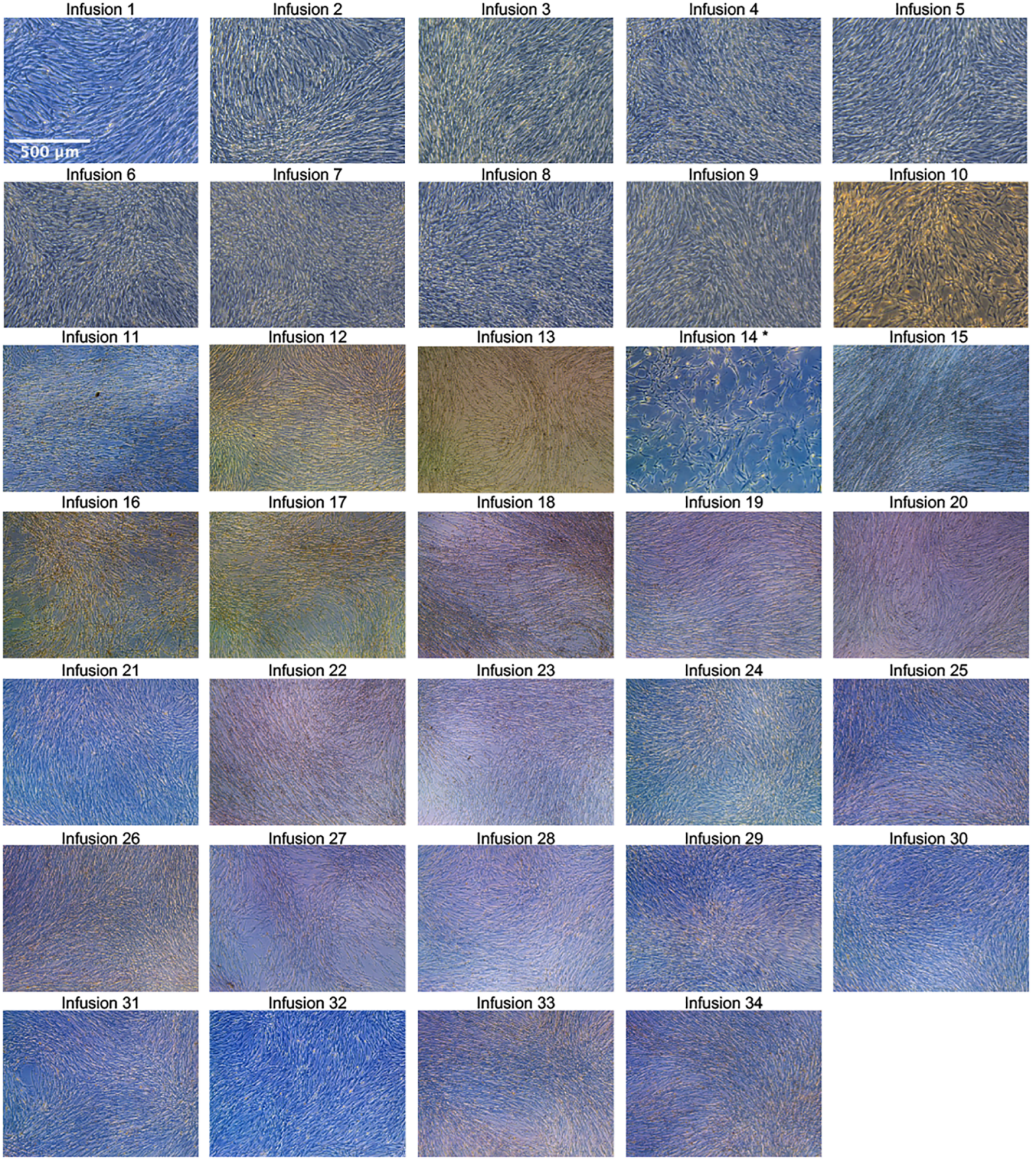
Passage 4 culture images for all 34 infusions. Images were taken with a Leica inverted microscope at 50× magnification. Color variation is due to flask wall thickness, angle, and light. * The product released for infusion #14 was lower than the minimum dose requirement (200 million live cells ±20%) due to the product material harvested at sub-confluency.

For the isolation of HB-adMSCs, first fat extraction was performed by a licensed physician from the patient's abdomen. The extract was then tested by the quality control unit at Hope Biosciences LLC., for USP71 sterility and mycoplasma due to possible contamination from the fat extraction procedures, followed by centrifugation to phase-separate the adipose tissue. A total of 7 ml adipose tissue was then treated with collagenase to isolate stromal vascular fraction (SVF). Cells from the SVF were plated in Hope Biosciences' HB-103 medium to establish a P0 culture. The resulting adherent cells were further cultured with HB-101, Hope Biosciences' growth medium. Cells were cryopreserved at passages #0, #1 and #2 to create a complete cell bank for the patient and an aliquot of #2 culture supernatant was cleared by the quality unit for USP71 sterility, mycoplasma, and endotoxin. For infusions, passage #2 cells were thawed, recovered in passage #3, and cultured to passage #4 (Figure 1). A total of 34 infusions (manufactured from the cell bank created for the patient and freshly harvested from passage #4), each with 200 million ± 20% MSCs mixed in 250 ml of 0.9% sterile sodium chloride were administered intravenously, over a period of ∼2.5 years (Table 1).

**Table 1:** Infusion details for all 34 infusions with MSC quality control metrics.

Infusion #	Date of administration (month/day/year)	Total cell count (million)	Cell viability (%)	CD73 (%)	CD29 (%)	CD31 (%)	CD45 (%)
1	10/11/19	205	98.46	98.69	99.97	0.24	0.10
2	10/25/19	224	93.33	96.86	99.95	0.22	0.04
3	11/08/19	240	94.25	97.90	99.92	0.07	0.08
4	11/22/19	178	94.87	98.12	100.00	0.10	0.11
5	12/06/19	166	94.55	99.47	99.99	0.17	0.16
6	12/20/19	186	98.31	94.82	99.96	0.05	0.15
7	01/3/20	195	96.06	97.66	99.96	0.04	0.06
8	01/17/20	200	96.03	90.17	99.92	0.09	0.09
9	01/31/20	131	97.62	99.00	99.96	0.14	0.08
10	02/14/20	232	98.64	98.50	99.91	0.31	0.05
11	06/24/20	240	95.18	95.01	99.69	2.16	0.07
12	07/24/20	240	95.24	93.89	99.97	0.06	0.00
13	08/21/20	224	95.89	96.23	99.88	0.00	0.03
14	10/02/20	99.2^a^	95.38	99.62	100.00	0.00	0.04
15	10/30/20	240	95.35	97.11	99.87	0.00	0.07
16	11/13/20	240	94.50	94.99	99.73	0.20	0.00
17	12/11/20	240	98.28	98.11	99.89	0.00	0.00
18	01/08/21	240	97.66	95.87	99.90	0.00	0.02
19	02/05/21	240	98.32	92.64	99.91	0.02	0.07
20	03/05/21	211	95.65	88.89	99.87	0.00	0.00
21	04/02/21	240	96.67	83.57	84.62	2.28	0.51
22	04/30/21	240	95.63	98.21	99.89	0.02	0.15
23	05/28/21	240	96.91	95.32	99.88	0.00	0.16
24	06/25/21	182	95.00	92.04	99.40	0.02	0.05
25	07/23/21	240	99.29	99.28	99.90	0.00	0.34
26	08/20/21	240	94.53	98.63	99.91	0.00	0.24
27	09/17/21	240	98.86	94.52	100.00	0.05	0.02
28	10/15/21	240	96.49	91.01	99.87	0.02	0.05
29	11/12/21	205	96.97	97.88	99.94	0.00	0.45
30	12/10/21	240	96.30	95.52	99.86	0.00	0.24
31	01/7/22	221	98.57	99.66	99.72	0.00	0.19
32	02/23/22	240	98.95	96.37	100.00	0.00	0.85
33	03/09/22	240	96.70	98.87	100.00	0.00	0.79
34	04/29/22	205	96.94	99.52	99.97	0.00	0.19

^a^ Low cell-count due to cell growth. MSCs are expected to be positive for CD73 and CD29 and negative for CD45 and CD31 cell surface markers.

Before administration of cells, each infusion product underwent cGMP-compliant quality control standard assessments, which included viability; appearance; sterility; gram staining; mycoplasma; endotoxin; and cell identity/purity as indicated by MSC defining surface markers to ensure a standardized product is delivered for each treatment. All HB-adMSCs were positive for CD73 and CD29, and negative for CD45 and CD31 (Table 1).

### 2.3. Results

Since the start of HB-adMSC therapy on October 11, 2019, improvements were seen in the subject's dexterity of fingers and hands, with observed improvements in his hand grip. Following the first few infusions, the subject began to operate his modified vehicle that eliminated his need to depend on his family for travel. He continued to show progress with the sustained MSC therapy and showed remarkable improvements in his quality-of-life. His sensation for bladder awareness improved which allowed him to catheterize himself based on need, as opposed to every 4 h previously. Also, his sensation for defecation improved which eliminated incontinence accidents and the use of suppositories that he needed in the past. With recurrent HB-adMSC treatments, he showed considerable improvements in his hands and arms with some improvements in his left leg that had led to a change in his condition from a quadriplegic to a partial paraplegic. The subject began to have increased sensation in his trunk and thighs and was able to voluntarily lift his left leg 8–10 inches from the wheelchair foot at the knee, dorsiflex his left foot, and push up on his toes. He was also able to wiggle the toes of his right foot and was able to go fishing (raising his arms over his head to cast). Post-intervention, the subject discontinued all his medications, and he could even stand without support for brief periods of time.

#### 2.3.1. SCIM-III scores

**Figure 2:**
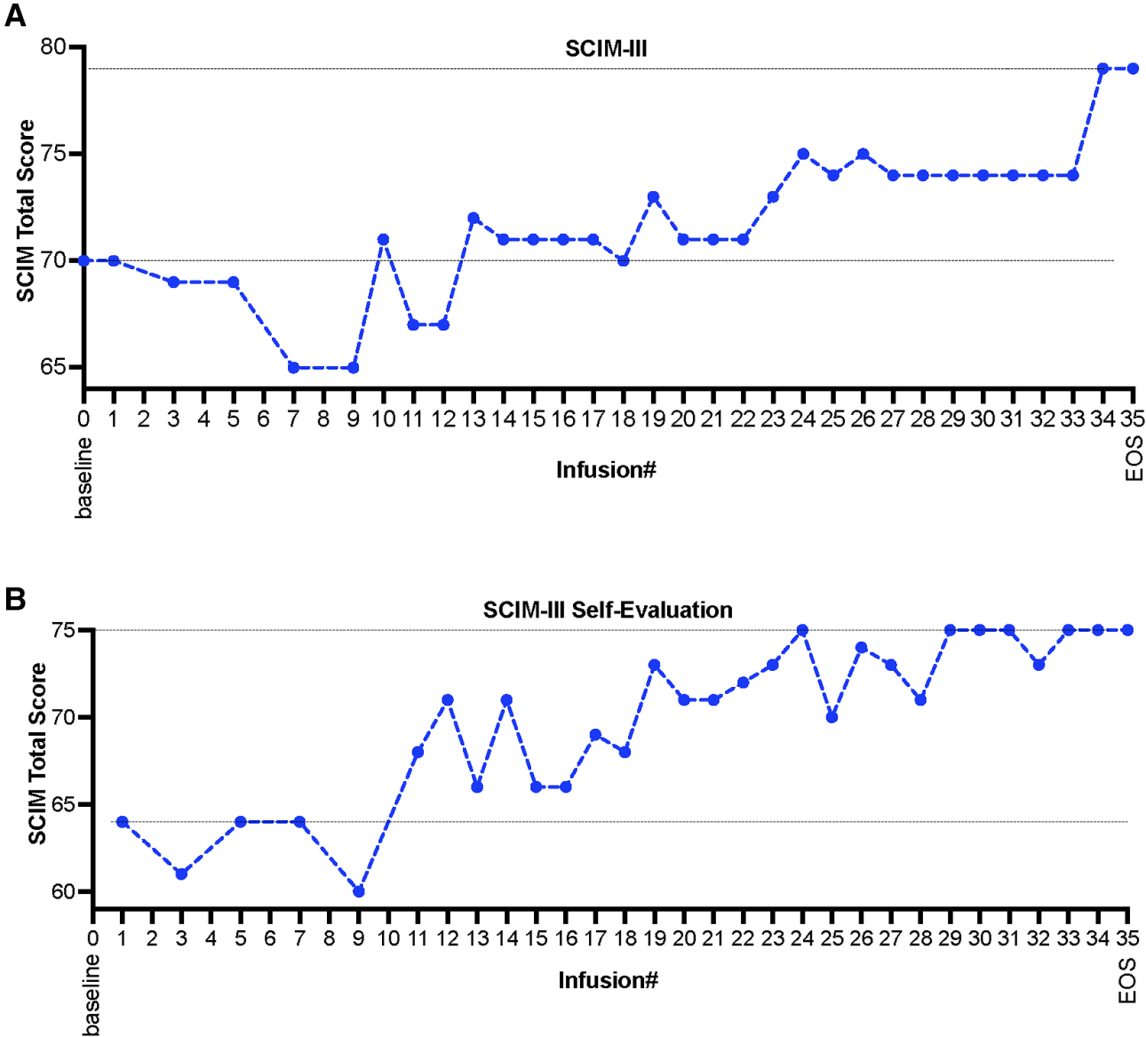
(**A**) SCIM-III scores as evaluated by the investigator, and (**B**) subject's self-evaluation assessment, measured at multiple infusion timepoints and at the end the study, after administration of 34 HB-adMSC infusions. SCIM-III, Spinal Cord Independence Measure III; EOS, end of study.

Evaluation of changes in the patient's ability of daily living was assessed using a clinician administered spinal cord injury disability scale, Spinal Cord Independence Measure (SCIM-III). SCIM-III scores were calculated using both investigator's assessments as well as patient's self-assessments at multiple visits during the whole treatment period. Measures of the SCIM scores demonstrated functional improvements as early as after the administration of ∼10–12 HB-adMSCs infusions (Figure 2). As per investigator's SCIM-III assessment, the subject started at a score of 70, that increased to a score of 79 by the end of the study (EOS), after administration of 34 intravenous infusions of HB-adMSCs (∼13% increase from the baseline; Figure 2A). Similar improvements were observed in the subject's self-evaluation SCIM scores (∼17% increase from the baseline; Figure 2B).

#### 2.3.2. Magnetic resonance imaging (MRI), electromyography (EMG) and nerve conduction velocity (NCV)

**Figure 3:**
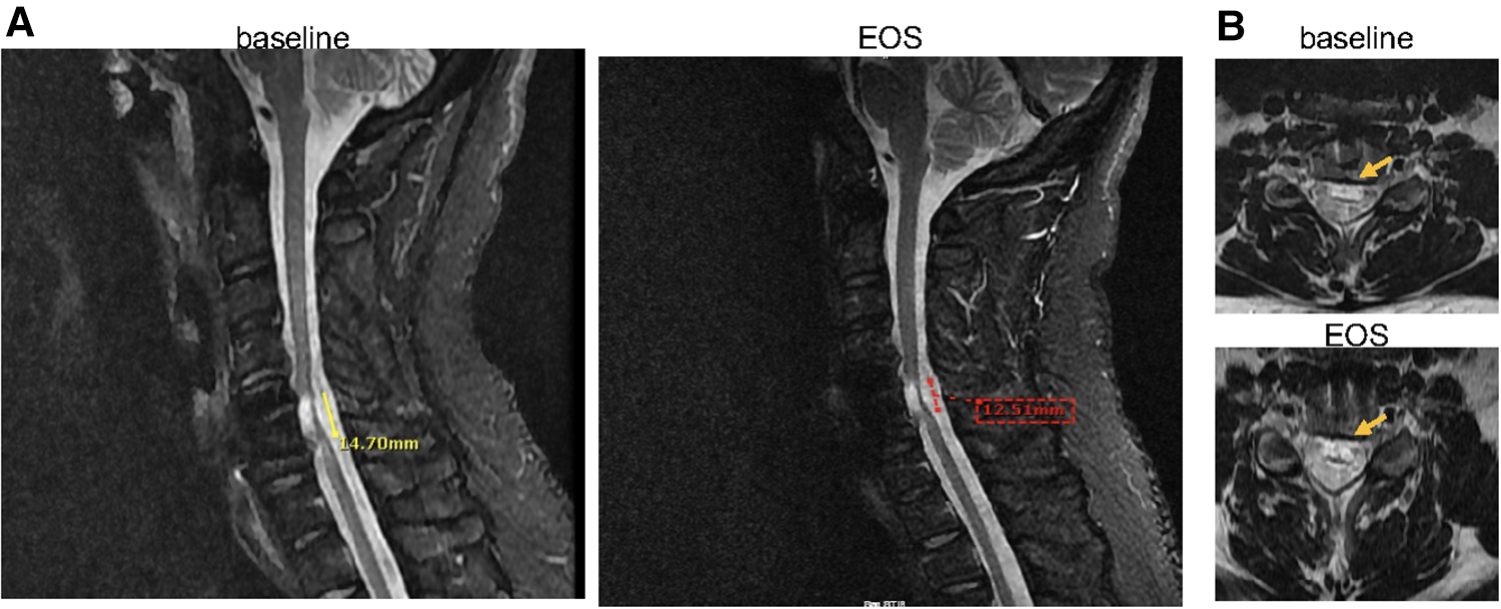
(**A**) Sagittal MRI images (baseline vs. EOS) shows mildly decreased craniocaudal length of the area of T2 abnormality (14.70 mm vs. 12.51 mm), and (**B**) axial view (baseline vs. EOS) shows minimal increase in the non-edematous portion of the cord at the EOS (post 34 infusions, at month 30) compared to baseline (represented by arrows). EOS, end of study.

To visualize the progression of the spinal cord injury over the course of the treatment period, MRIs were conducted at baseline, after infusion #17, and at the end of the study (EOS)-after infusion #34. Vertebral fusion at C5–C6 with central disc herniation at C4–C5 was reported in all MRIs with no significant changes at the EOS compared to baseline. Moreover, hypersensitivity in a lesion at C5–C6, indicative of myelomalacia, was reported in all MRIs. Although no significant changes were observed between MRI reports at baseline and at EOS, however, there might be an indication of subtle improvement. As observed on the sagittal images, there was a slight decrease in craniocaudal length of T2 abnormality at the EOS, by approximately 2 mm, when compared to baseline (Figure 3A). Also, on the axial images (Figure 3B), there is a suggestion that the non-edematous cord was slightly thicker at the EOS compared to baseline, although this evaluation is limited by the limited number of axial slices through the lesion on both exams.

To access functional changes in the recovery of voluntary movements, needle electromyography (EMG) and Nerve Conduction Velocity (NCV) studies were performed at multiple time points: infusion#1 (baseline) and subsequently after infusions #11, #17, #23, #29, and at EOS. The initial EMG/NCV report at baseline (Infusion #1) revealed electrophysiological abnormalities, including moderate chronic axonal injury at the C5–C6 and C6–C7 nerve roots bilaterally, moderate chronic right median motor and sensory neuropathy from the wrist to palm, and mild ulnar motor neuropathy in cubital tunnels bilaterally. Specifically, there were decreases in conduction velocities noted in right median motor nerve, left and right ulnar motor nerves, right median sensory nerve, and left sural sensory nerve (details of specific motor and sensory nerves with decreased conduction velocities are provided in Supplementary Table S1). However, after the first 11 infusions of HB-adMSC therapy, significant improvements emerged in conduction velocities of the left and right ulnar motor nerve (increasing from 38 to 59 m/s and 50 to 69 m/s, respectively) and in the right median motor nerve (increasing from 48 to 59 m/s) (Supplementary Table S1). Additionally, there were improved amplitudes observed in the left and right tibial motor nerves (1.8–8.4 mV and 1.4–5.2 mV, respectively). Similarly, increased conduction velocities were noted for the right median sensory nerve (38–44 m/s), and for left sural sensory nerve (24–38 m/s). Improvements continued to be observed after infusion #17, with additional progress noted in conduction velocities of the sensory sural nerve (38–61 m/s). By the end of the study (after administration of 34 infusions, in total), left ulnar motor nerve action potentials and conduction velocities were within normal limits. However, for the right median and ulnar motor action potentials at the wrist and forearm, a slight prolongation was observed after initial improvements, possibly attributed to intensive physical therapy that the patient underwent during that period. While improvements in bilateral lower extremities waned between infusions 23 and EOS, marked improvements were evident in left sural and peroneal sensory nerve action potentials. Additionally, improvements in distal latencies were also documented in these reports.

To assess the safety of the intervention, we performed standard laboratory evaluations of complete blood count (CBC), comprehensive metabolic panel (CMP), and Coagulation Panel (CP) at multiple timepoints during the entire duration of the study. Analysis of the laboratory values for any of the CBC, CMP or CP components did not exhibit any unusual changes, when compared to the values at baseline. No treatment-related serious adverse events were reported during the entire course of treatment. Only a few adverse events were reported during the study period that included cough, fatigue, dizziness, bursitis, COVID-19, a fall with injury, and a headache that were all mild in severity, and none of these were related to the intervention.

## 3. Discussion

Currently, no treatments exist that can fully restore the injury-induced functional loss caused by SCI. Owing to their neuroprotective and axon-regenerative potential, MSCs offer a promising therapeutic avenue for the treatment of SCI. Several mechanisms have been proposed by which MSCs are known to ameliorate SCI, including secretion of neurotrophic factors such as brain-derived neurotrophic factor (BDNF), that has been suggested to be a part of the mechanism underlying rapid functional recovery in pre-clinical models of SCI (18, 19). More specifically, adMSCs have been demonstrated to promote cell survival and tissue repair by increasing the expression of neurotrophic factors as shown in animal models of SCI (1, 20). Aside from being an attractive MSC source due to their easy availability and strong proliferative capacity, adMSCs also exhibit relatively improved neuro-regenerative potential in improving functional outcomes of SCI, as indicated by preclinical studies (21). Although clinical evidence for the safety of adMSC remains limited (13, 14), multiple pre-clinical studies demonstrated safety of adMSCs in animal models of SCI (20, 22). Furthermore, clinical implication of safety of MSCs derived from various other sources for the treatment of patients with SCI had been previously suggested, without any indications of any significant adverse events observed across multiple routes of administration (15, 23–25).

Therapeutic potential of MSCs derived from multiple sources has also been demonstrated in several clinical studies for the treatment of SCI, but with less consistent clinical outcomes (13, 26, 27). Only a few clinical trials demonstrated the efficacy of adMSCs in ameliorating SCI, with improvements in motor and sensory function. Hur et al. (13), employed intrathecal administration of autologous adMSCs in 14 SCI patients and the results of the study showed motor improvements in five patients with improvements in sensory function in ten patients. Recently, Bydon et al. (14) employed intrathecal injection of adMSCs and reported improvements in motor and sensory scores, based on International Standards for Neurological Classification of SCI. Here, in this study, we administered multiple intravenous infusions of autologous HB-adMSCs and showed substantial improvements in our SCI patient, improving his overall quality-of-life. In the current study, we used SCIM-III, one of the sensitive tools for monitoring functional recovery in SCI patients (28). Our results demonstrated a gain of ∼10 points in SCIM-III score, implicating clinically relevant functional improvements in this SCI patient. Additionally, comparison of the EMG and NCV studies pre- and post-therapy demonstrated substantial improvements in the patient's motor and sensory function. These electrodiagnostic studies conducted at various study timepoints showed improvements in conduction velocities, distal latencies, and amplitudes of the peripheral nervous system, indicative of axonal regeneration and re-myelination of the demyelinated nerve fibers due to SCI.

Although a large focus of myelomalacia were reported in both the baseline and EOS MRI, these reports showed no evidence of injury progression, suggesting that the treatment with HB-adMSCs was safe, with no new abnormalities observed post-treatment. Changes in cell-dose, administration route, or longer follow-up period may be required to detect gross anatomical and structural changes in MRI. However, at least another study using intrathecal administration of autologous adMSCs showed no interval change in lesion site between baseline and at the 8-month follow-up MRI, post-treatment (13). Moreover, while MRI has become a conventional imaging technique for spinal cord injuries, it has been suggested that its usage may be limited because of the non-specificity of changes in signal intensity that do not directly reflect physiological changes (29). Additionally, there is evidence that MRIs are more effective when used acutely following a spinal cord injury, specifically within the first 72 h, for prognostication, as opposed to being used for management over lengthy durations of time, as was done in this study (30). These aspects should be considered while interpreting such results. Nonetheless, neither this study nor another clinical study that employed intrathecal adMSC therapy in treatment of SCI (13) presented any serious adverse events, implicating the safety of adMSCs via at least two different routes of administration.

This study has a few limitations. The assessment of spinal cord injury progression relied on MRI, which exclusively captures structural changes and does not reveal any functional alterations post-therapy. In order to enhance sensitivity in monitoring physiological changes resulting from therapeutic interventions for SCI, other advanced imaging techniques such as SPECT (Single Photon Emission Computed Tomography) or PET (Positron Emission Tomography) should be considered to quantitatively evaluate functional aspects of the recovery process, thereby providing a more comprehensive assessment of therapeutic efficacy. Additionally, the study protocol did not incorporate ASIA score assessments—a standardized tool used to evaluate and classify the severity of spinal cord injuries based on sensory and motor function. Despite there were noticeable improvements in the subject's sensory and motor functions, as indicated by the classification shift from ASIA B C-5 to T-5 in the Principal Investigator's notes, the absence of ASIA assessments may have limited a more precise reflection of these improvements.

## 4. Conclusions

Overall, HB-adMSC therapy was safe and effective in improving the patient's neurological function and thus quality-of-life. As demonstrated by our case report, recurrent administration of multiple intravenous infusions of autologous HB-adMSCs resulted in improved SCIM-III scores with improvements in motor and sensory function, as depicted in electrophysiological findings performed during the treatment period. HB-adMSC therapy may offer a relatively safe and non-invasive treatment option with a potential to improve neurological function of patients with SCI. The results of the current study suggest that sustained continual treatment may result in further clinical improvements. Additionally, future clinical trials with a larger sample size, using a randomized, placebo-controlled study design should be conducted to validate the findings of this study.

## Data Availability

The original contributions presented in the study are included in the article/**Supplementary Material**, further inquiries can be directed to the corresponding author.
