# Supplementary material for: Functional recovery of a 41-year-old quadriplegic spinal cord injury patient following multiple intravenous infusions of autologous adipose-derived mesenchymal stem cells: a case report
Source: Front Transplant. 2023 Dec 7;2:1287508. doi: 10.3389/frtra.2023.1287508 (PMC11235215; doi:10.3389/frtra.2023.1287508)
Supplement: Supplementary file 1 [file Table1.docx]

**Table S1. Nerve Conduction velocity (NCV) data for specific motor and sensory nerves that exhibited decreased velocities at**

**baseline (Infusion #1).**

| **MOTOR NERVE** | **Infusion #1** | | **Infusion #11** | | **Infusion #17** | | **Infusion #23** | | **Infusion #29** | | **EOS** | | **Normal** | |
| --- | --- | --- | --- | --- | --- | --- | --- | --- | --- | --- | --- | --- | --- | --- |
| Right Median | 48 | | 59 | | 51 | | 54 | | 54 | | 50 | | >50 | |
|  |  | |  | |  | |  | |  | |  | |  | |
| Left Ulnar | 38 | | 59 | | 43 | | 54 | | 51 | | 54 | | >53 | |
| Right Ulnar | 50 | | 69 | | 75 | | 63 | | 60 | | 53 | | >53 | |
|  |  | |  | |  | |  | |  | |  | |  | |
| **SENSORY NERVE** | |  | |  | |  | |  | |  | |  | |  |
| Right Median | 38 | | 44 | | 44 | | 42 | | 40 | | 41 | | >39 | |
|  | | | | | | | | | | | | | | |
| Left Sural | 24 | | 38 | | 61 | | 33 | | 29 | | 48 | | >35 | |

At baseline (Infusion #1), decreased conduction velocities were observed for motor (right median, left ulnar, and right ulnar) and sensory (right median

and left sural) nerves (conduction velocities for all other motor and sensory nerves were within normal limits at the baseline). Post-therapy, improvements

in conduction velocities were seen at multiple time points over the course of the study. EOS, end of study.
